# Living with polypharmacy: a narrative interview study with older Pakistanis in East London

**DOI:** 10.1186/s12877-023-04392-1

**Published:** 2023-11-15

**Authors:** Najia Sultan, Deborah Swinglehurst

**Affiliations:** Wolfson Institute of Population Health, Queen Mary University of London, London, UK

**Keywords:** United Kingdom, Narrative methods, BNIM, Polypharmacy, Immigrants and migrants, Qualitative

## Abstract

**Background:**

Polypharmacy is a growing and major public health issue. It can be burdensome and risky for patients and costly to healthcare systems. Older adults and those from ethnic minority backgrounds are disproportionately affected by polypharmacy. This study focuses on medication practices among Urdu-speaking Pakistani patients, a significant ethnic group in the UK. Most existing research on medication practices within South-Asian communities centres on adherence, leaving the social and moral dimensions of polypharmacy unpacked. Understanding how British Pakistani patients understand and manage polypharmacy in the context of their daily lives is crucial to avoiding harmful polypharmacy.

**Methods:**

In-depth narrative interviews were conducted with 15 first-generation Pakistani patients using the Biographical Narrative Interview Method. Participants were recruited from GP practices in East London. All participants were prescribed ten or more regular medications (a pragmatic marker of ‘higher risk’ polypharmacy) and were aged over 50. Interviews were conducted with a bilingual researcher at home and were designed to elicit narratives of patients’ experiences of polypharmacy in the context of their biographies and daily lives.

**Results:**

Polypharmacy is enacted through networks of interpersonal and socio-material relationships. The doctor-patient relationship and the family network held particular significance to study participants. In addition, participants described emotional bonds between themselves and their medicines, identifying them as ‘forces for good’—substances which allowed them to maintain their health through the intercession of God. Meanings attributed to medicines and enacted through these social, emotional, and spiritual relationships contributed to emerging and sustaining polypharmacy.

**Conclusions:**

Patients make sense of and manage treatments in culturally specific ways. Developing an understanding of how medication practices in different communities are enacted is important for informing meaningful and effective conversations with patients about their medicines. Our findings contribute to enabling the integration of culturally sensitive approaches to prescribing.

## Background

Polypharmacy is a growing and major public health issue [1]. In England, 8.4 million people are prescribed more than five regular medications (‘polypharmacy’) with a quarter of adults aged over 85 prescribed eight or more medications regularly [2]. Polypharmacy disproportionately affects ethnic minority communities and older adults and is thus one expression of health inequality [3, 4]. Though polypharmacy is sometimes necessary, managing polypharmacy can create a burden for patients. Polypharmacy can also cause iatrogenic harm, the risks increasing both with age and the number of medications prescribed [5]. In a healthcare system under considerable pressure, polypharmacy is resource intensive. Prescribing represents 16% of the NHS budget and adverse drug reactions constitute up to 6–7% of hospital admissions in people over 65 [6].

Escalating polypharmacy is usually attributed to the ageing population who are susceptible to multiple long-term conditions. This is a partial account of the problem. Critics argue that the organisation of care, guidelines and pay-for-performance policies based on the ‘single disease’ model also drive and sustain polypharmacy, prioritising concerns about diseases over a focus on the person in their lived context [7–9]. Scholars have described deprescribing as ‘swimming against the tide’ of patient expectations and normative medical culture. There are many barriers and few incentives for deprescribing as part of routine care [10].

Building an understanding of the factors that contribute to polypharmacy in migrant groups is complex. Cultural backgrounds impact both health-seeking behaviours and patients’ relationships with medications. Migrants may have different health needs and different expectations of healthcare systems to those of native populations [11]. Furthermore, influences from migrants’ countries of origin may lead to supplementary use of health care services from abroad [12]. Language barriers and the absence of support networks may also underpin and exacerbate chronic conditions and associated polypharmacy [13]. Those of lower socioeconomic status have higher odds of experiencing polypharmacy; and the prevalence of prescribed medicine use is higher in deprived areas, where ethnic minority groups often live [14, 15]. It can be difficult to disentangle the influences of ethnicity, culture, inequalities and deprivation on migrants’ experiences and understandings of medicines.

Despite many migrant groups being disproportionately affected by polypharmacy, most previous research exploring peoples’ experiences of living with polypharmacy has focused on participants who speak English [16]. Polypharmacy is rarely addressed through a lens which accommodates an understanding of what it means to ‘live’ it within a social and cultural context. There is a dearth of research which opens the possibility for participants to articulate, in their own language, the lived experience of polypharmacy.

### Medicines and Pakistanis

Britain is home to 1.17 million Pakistanis, the largest Pakistani community in Europe. Urdu is the national language of Pakistan and the UK’s third most commonly spoken immigrant language [17]. Over 90% of Pakistanis identify as Muslim. Pakistani family units are closely knit; extended families frequently live together in multi-generational family homes or are based locally. British Pakistanis experience high levels of deprivation and poverty; 47% of British Pakistani households are in the lowest income quintile, the highest percentage of all ethnic groups [18]. Pakistanis also bear an excess and premature toll of chronic illness compared with other ethnic groups in Britain, especially from cardiometabolic conditions including heart disease, diabetes, and hypertension [19]. Cardiometabolic conditions are notable for associations with high rates of polypharmacy [20].

Existing evidence suggests that Pakistani migrants have specific cultural beliefs about how and where to access medicines and the importance of medication use. In Pakistan, there is a large informal healthcare sector with a high prevalence of self-medication and poorly regulated drug-to-consumer drug marketing. One Norwegian interview study with 82 first-generation Pakistani migrants found that the large majority believed it was important to take medication every day [21]. In a study of self-medication amongst Pakistani mothers in New Zealand, 87% of participants reported having brought medicine, including antibiotics, into New Zealand from Pakistan during their last trip. Participants cited concerns about not being able to get medication when they wanted it as reasons for bringing medications from ‘back home’ [12]. Whyte et al. note that we live in a globalised world: “medicines not only move, as they always have done to some extent but where they move has implications about influence, dependence and transformation” [22]. Thus, whilst first-generation mothers from New Zealand travelled *from Pakistan* with medication, first-generation Norwegian Pakistanis described the importance of travelling with medications *from Norway* for trips ‘back home’. For older Norwegians Pakistanis, medicines from Norway were deemed to be more trustworthy than those obtained from Pakistan, in part because they were less likely to be generic substitutions. Studies exploring this phenomenon have shown that generic substitutions are a challenge to drug adherence amongst first-generation Pakistanis, attributed to participants’ heightened awareness of counterfeit drugs from their native country [23]. Better understanding the complex ways in which many Pakistani migrants make sense of and manage their medicines is a crucial first step in addressing polypharmacy in this population.

## Study purpose

We used narrative methods to study how older British Pakistani patients articulate their lived experience of polypharmacy. Our research questions were:

- What is the patient experience of polypharmacy?
- How do patients manage their medicines in the context of their daily lives?
- How do patients describe their relationships with professionals involved in their medicines management?

## Methods

### Study design and setting

This study was undertaken in the neighbouring East London boroughs of Newham and Tower Hamlets; two of the UK’s most densely populated and deprived local authority areas with large Pakistani communities and visible Muslim populations. Research has shown that multimorbidity occurs 10 – 15 years earlier in the most deprived areas in the UK, with significantly higher rates of physical–mental comorbidities [24]. Fifteen people were recruited through seven GP practices. The inclusion criteria were: patients aged over 50 (to reflect the early onset of multimorbidity in this population) of Pakistani ethnicity who spoke Urdu or were bilingual Urdu-English speakers, with two or more ongoing medical conditions and prescribed 10 of more items of regular medication, an indicator for higher risk polypharmacy [25]. A nominated GP in each practice conducted an electronic search to identify eligible participants, sent invitation packs in Urdu & English, and sought verbal consent from interested potential participants for a researcher to contact them by telephone to explain the study. Sampling was purposive to identify maximum diversity. The researcher, NS is a Pakistani female doctor working in general practice. She introduced herself as a university researcher. None of the research participants were recruited from the GP practice in which NS works.

### Ethical considerations

The study was granted a favourable ethical opinion by North East Tyne & Wear South Research Ethics Committee (IRAS project ID: 228,870; REC reference 17/NE/0314) via proportionate review.

### Data collection

Interviews were conducted based on an adapted version of Wengraf’s Biographical Narrative Interpretive Method (BNIM). BNIM interviews start with the use of “single question aimed at inducing narrative” (SQUIN) [26]. The SQUIN was*: Can you please tell me your story of your life since you were first advised to take medicines. I would like you to tell me about all the experiences, people, and events, which are important to you personally. Please begin wherever you like and take as long as you need.* Participants were invited to respond to this initial question uninterrupted whilst the researcher made notes and identified cue phrases. The interviewer then used participants’ cue phrases to elicit more detailed narratives. Finally, a short semi-structured interview was conducted using an interview topic guide, designed to enable clarification and address gaps relevant to the research question. Observational field notes were made following the interview. Interviews took place between December 2017 and June 2018 and lasted a mean of 72 min. Interviews were audio-recorded, transcribed, and translated into English by NS*.*

### Analysis

Narrative interviews offer a way of collecting people’s stories about their experiences with health and care. Scholars have suggested that the inherent subjectivity of narrative interviews means this method is particularly well-suited to examining the perspectives of disadvantaged groups such as minorities, the seriously ill or old [27]. We followed Muller’s four steps of narrative data analysis here: data entry (reading, sorting to gain familiarity), sense-making (finding connections, patterns through successive readings and reflection), verifying (searching for alternative explanations, confirmatory and disconfirming data) and accounting for what has been learnt [28]. Transcripts were initially coded manually and then on NVivo where coding categories and themes were organized and discussed between the authors. Three data workshops involving members of the multidisciplinary Apollo research team were used to develop and refine our analysis [29]. A reflexive research journal was kept throughout. This study is reported in line with COREQ guidelines for qualitative research.

## Results

Fifteen in-depth narrative interviews were conducted in participants’ homes, 13 in Urdu and 2 in English. Patients were invited to have someone with them at the interview if they wished and 5 participants chose to have family present, either spouses or adult children. Patients were on 10—15 separate items of regular medications and had a wide range of health conditions including diabetes, hypertension, asthma, thyroid, and mental health disorders. All participants were first-generation Pakistani migrants. Demographic characteristics can be viewed in Table 1.

**Table 1:** Demographic characteristics of study participants

**Variable**		**Number of Participants**
Age	50–64	4
	65–80	9
	80 +	2
Gender	Male	6
	Female	9
Time in UK	0–9 years	0
	10–19 years	5
	20 years +	10
Number of medications	10–12	7
	13–15	8

### Overview of results

The overarching finding was that polypharmacy is created and sustained through a network of interpersonal and socio-material relationships. Within interpersonal relationships, the doctor-patient relationship and the family network held particular significance to our participants. On a socio-material level, our participants frequently described the emotional relationships they formed with medications as objects that are a ‘force for good’ in their lives and substances which allow them to maintain their health via the intercession of God *(“I ask God for my medicines and my doctors deliver them to me”*). These relationships existed before, beyond, and often despite an understanding of the technical or biomedical properties of their medicines. Meanings attributed to medicines and enacted through these social, emotional, and spiritual relationships contributed to emerging and sustaining polypharmacy.

We will present our findings as two themes; firstly, we consider the interpersonal relationships that create and sustain polypharmacy including that of the doctor-patient relationship and the role of the family network. Our second theme focuses on the socio-material relationship participants form with medicines themselves, as objects of spiritual relevance and material ‘forces for good’.

#### Interpersonal relationships and polypharmacy

### Good doctors prescribe medicines and good patients accept medicines

Throughout our interviews, participants related the role of the doctor as an advisor, prescriber, and provider of medications. This 72-year-old female participant describes an encounter with her GP where she was advised to continue taking blood pressure medications:

Interviewer: You also mentioned that you must take your medicines?

14: Yes, I must. Otherwise, what is the point of visiting the doctors? If I did not want to take the medicines, I would not visit the doctor.

She suggests, in a way which conveys a ‘taken-for-granted’ understanding, that receiving medicines is ‘*the point’* of visiting a doctor. Throughout our interviews, participants often described receiving a prescription as a central outcome of the doctor-patient consultation. In the following data extract, this 77-year-old man associates doctors with *‘good habits’* as those who *‘give good medicines.’* He goes on to identify himself as someone who has (under his doctor’s guidance), *‘taken so many medicines’,* suggesting his part as a good recipient of medicines. Medicines are thus used as a tool, to construct to the listener, how *‘good’* both he and his doctor are in their respective roles, foregrounding the implied moral contract within the doctor-patient relationship:

The doctors would check us and tell us what medicine we needed for our illness and for how long … They check your blood pressure properly. They ask you questions, where’s the pain and things. They check your eyes. They check your ears. They look at everything. Then they give you medicines… If I needed to go to get medicines, (Dr Jones) would sit me down with great care and listen to what I had to say with great care. He would give you medicines with great care … Dr Jones is good to everyone. He has good habits. He gives good medicines. He speaks well to people. He is always good to us. Whenever we go to see him, straight away he gives us medicine… I have taken so many medicines. He has given me so many medicines, Dr Jones. He really has listened to us. He is a very good man [10]

Note how a visit to the doctor is described by the participant as when he ‘*needed to go to get medicines’.* The participant takes care to present his doctor as a moral individual, using an extreme case formulation as a rhetorical device to achieve this: his doctor *‘look(s) at everything’* and is *‘good to everyone’* [30]. He also constructs a close alignment between how ‘*good’* a doctor is and the issuing of a prescription. Lastly, note how the participant associates being given *‘so many medicines’* with being *‘listened to’.*

Participants in this study consistently verbalised high levels of confidence in the prescriptions they received from their doctors. Although we also sought to elicit concerns or criticisms these were very rare and focused almost entirely on difficulties accessing appointments or time constraints within consultations rather than on prescribing or medicines per se (‘*Doctors are always in a rush now’*). The notable absence of more critical narratives around prescribing and medicines became a key interest in our iterative enquiry and analysis. We reflect on this finding and our interpretations of it below. First, here is a short illustrative extract from an interview:

Interviewer: Have you ever had side effects because of your medicines?

10: No, no, no. We get good medicines.

Interviewer: What do you mean by that?

10: I mean he (the doctor) gives us good medicine, the right medicine. He doesn’t give us the wrong medicine. It is only if someone gives you the right medicine that you will get relief … Yes, the medicines that will suit the body, our doctor gives those.

Note the emphatically repeated *‘no’* on three occasions when asked about side effects, followed by the description of his medicine as ‘*good’*. There is an assumption here that the doctor would not give a medicine with a side effect, a display of considerable trust in both the drugs and the doctor. Within this quote, moral descriptors are repeatedly attached to medicines which are described as being *‘good’, ‘right’* or *‘wrong’.*

Participants took care to construct narratives of themselves as good, compliant, and agreeable patients, like this 72-year-old female participant who takes ‘*whatever’* she is given by her doctors:

Interviewer: When the doctor gives you medicines, what is the most important thing you think about?

4: I do not think about anything! I just think, what I am being given I must just take it. I do not think why they are giving it or what they are giving. Whatever they give me, I take it.

Medication adherence is thought to be particularly problematic in the South Asian community [31–33]. We were surprised at the *absence* of narratives of ‘non-adherence’. This was especially remarkable in the context that our interview participants displayed a considerable willingness to discuss topics that might be regarded as sensitive, for examples see [34]. Our data set included no reports of non-adherence or criticisms of doctors’ prescribing decisions, despite these issues being explicitly explored. Interviews were also devoid of any critique of wider structural factors that may drive polypharmacy. In the following extract from her reflexive journal, the first author considers the tensions that she experiences in her dual role as clinician and researcher.

**Table:** 

As a doctor working in East London, I am surprised by the contrasting positivity with which participants repeatedly speak of their doctors and their medicines in the interviews I am doing, compared with what I see on the General Practice front line. I know from my clinical experiences that people frequently come in unhappy about previous medical consultations. They often want to discuss medications they do not think are working for them and express frustration at having to take even more medication. These sentiments are absent in my interviews.
My experience of adherence within the clinic is that patients almost never acknowledge purposefully not taking or wasting medication. If this is uncovered somehow, it is almost always justified as an occasional occurrence, mistake or error by an external party (e.g. ‘*the pharmacy keeps sending me this drug that I do not take’*). Some topics seem legitimate to discuss within the confidential ‘safety’ of a clinical consultation. These include topics which may be regarded as quite sensitive – such as disliking a previous doctor they have seen or certain medicines that I or someone else may have prescribed.
In the interview context, however, I encounter something different - research participants performing to a ‘stranger’ in the presence of a recording device. The participants in this study have all been recruited through their GP surgeries – a few of them even comment that I have been sent by their surgeries. In this context perhaps it is unsurprising that they are hesitant to criticise their GPs or their prescribing choices despite my assurances of confidentiality. Here I see participants constructing their identities as compliant patients who listen to their doctors and do everything that their doctors suggest. I wonder if this phenomenon is amplified in the Pakistani community where exhibiting high levels of respect for doctors is certainly the social norm.
*NS, extract from reflexive journal (June 2018)*

### Family networks supporting the work of polypharmacy

We have recently published work describing the central role of the family network in the health care of older British Pakistanis [34]. In this study, we focus on the role of the family network in managing polypharmacy. This 77-year-old participant lives alone with his wife, having migrated from Pakistani 27 years earlier. They are both prescribed over ten regular medications. Their four adult children live locally, visiting their parents twice a day to administer their medication.

10: Morning and night our kids give us our medicines. In the morning our daughter comes and gives us our medicines and in the night our son comes to give us our medicines. … We don’t just take them by ourselves. They know which medicines we need to and shouldn’t take… We are grateful that our children come and gives us our medicines. That they can read and write.

Interviewer: So, all your medicines are given by your children – and you never take your medicines by yourself?

10: No, never. I can’t read English. I don’t know what each tablet is…

Interviewer: Is there a particular child …

10: No, they all know… They’re good, we’re lucky our children are good.

The participant describes his children as giving him medicines ‘morning and night’, a loyal kinship that places the medicinal needs of the participant central to the daily lives of his children. Like the doctor-patient relationship we described above, the participant constructs a moral link between his access to medicines and the actions of *‘good’* people (‘*our children’*) who facilitate this. Within this family, the ‘work’ of taking medicines was thus highly collaborative and distributed over multiple relationships within the nuclear family, with no single individual holding ultimate control over how, why and when medications were used.

The efforts that must be made by families to support sustained medication use can place emotional and practical burdens on family members [35, 36]. The son of this 81-year-old participant attended all his mother’s doctor’s appointments and managed her care. He described having to frequently call the GP to obtain repeat prescriptions for his mother’s tramadol (an addictive painkiller and controlled drug), which were often declined due to over-requesting. The anger and frustration experienced by his mother then had to be managed by the wider family. The participant would express distress, her daughter-in-law would phone her husband, and the participant’s son would then need to spend time during his working days visiting the GP and making in-person requests for the tramadol to be re-issued. Whilst the family were aware of the health risks of tramadol*,* they also acknowledged that their mother had become addicted to this medication and without it, the stress on the family was difficult to manage. When we asked the participant as to why this scenario played out in this way, the participant explained that she would rather have her son speak with her doctors about medication requests than do it herself as doctors asked her ‘*so many questions’*:

Whenever I go (the doctors) ask me so many questions… they say your mum she takes too much tramadol … she does this, she does that … I just want them to give it to me normally [13]

Family members of another participant noted that when she attended an appointment and was not given medication for her condition, the participant would be agreeable during the appointment and then come home and question her family as to why the doctor had not given her a prescription. This role of adult children in obtaining medicines, administering medicine, and advising about medicine was a consistent finding throughout the interviews.

Interviewer: If by speaking to the doctors you could change anything about your medicines what would you change?

7: Very rarely I suggest something…otherwise I do not suggest anything myself. I leave it to the GP. Or I consult my son to ask him what I should do. Before I would advise my family for everything, now I consult them. I feel that I am not in the position to advise them, but I must consult them. I must ask them if it is okay…. I feel it because of age factor. I have got too old.

The ‘close’ involvement of family members in medication practices was not limited by geography. Family members who lived distant from participants were also actively involved. One participant described using an over-the-counter nutritional supplement called glucosamine for joint pains. She explained that the specific formulation that suited her was only available in Australia, where her daughter lived:

It has been 10 years since I have been taking this medicine…. because of this I am still able to walk. This comes all the way from Australia. When (my daughter) would come she would send me it with the latest expiry date. Recently my grandson’s son came (from Australia) and he brought me three boxes. I only had a few… May Allah (God) give my grandchildren a long time. Whenever they come here or Pakistan they bring my medicine [6]

Note the belief that *‘because of this [medicine] I am still able to walk’*, presented as a way of legitimising the medication being sent from Australia and the ongoing involvement of multiple generations of the family to maintain her supply.

### Socio-material factors in polypharmacy

#### Medicines and Muslims

All the participants in this study were Muslim, as are over 90% of the British Pakistani community. Within the Muslim faith, every part of every life of every human, who has ever or will ever exist, has been planned in minute detail by God; and unfolds in a way that is pre-determined, purposeful, and unique to that individual. Through this lens, all health and illness, including issues such as having access to care (or not), and treatment success (or failure) are mediated through and pre-determined by God’s will. By extension, most Muslims accept that the most effective medication for a health problem may not work for them should it not be willed by God. Conversely, a treatment that may be considered ineffective by scientific standards may sometimes offer a cure—dependent on God’s will. Previous scholars researching the British Muslim community have been “struck with the absolute faith which people expressed in Allah” in relation to healing and health [37].

The omnipotent presence of God is well articulated by this 77-year-old male participant as he reflects on daily life:

We spend our time sitting here- taking our medicines—watching TV (Laughs) God is watching us. God is watching [10]

He suggests that taking medicines and watching TV have become significant pastimes. ‘*God is watching’* he says laughing, implying that these routine everyday activities are mediated and observed by God. In the Muslim worldview, there is no aspect of life or death that is too small to exist beyond God’s determination: “*Not even a leaf falls without His knowledge, nor a grain in the darkness of the earth or anything—green or dry—but is written in a perfect record” (*Quran: 6:59).

In Islam, *shukr* (gratitude) and *sabr* (patience) are fundamental virtues that underpin Muslim wellbeing [38]. Note how this 70-year-old female participant reflects positively on being started on insulin injections after the birth of her child:

Interviewer: Have you ever found it difficult to take your medicines?

11: No, never. Never. I take it regularly and with great ease. I never think ‘Oh Allah why am I taking all these pills, what is happening?’. I never feel like this…Allah knows better, what is happening and why it is happening. … Now thanks to Allah it has been 30 years since I have been taking my medicines and injections. It has been 30 years and I have had 30 years of life, have I lost anything?

This participant links faith, health, and medicines, and reiterates the core Islamic values of patience and gratitude. She reflects that she *‘never’* thinks *‘why am I taking all these pills’*, thus verbalising her faith in God’s will and expressing gratitude for her ongoing health with a *‘thanks to Allah … [for] 30 years of life’.*

There is a long history of complementary faith healers practising within the British Muslim communities [39]. The following participant explained that he was himself a religious healer or *‘hakim’*. He describes his views on treatment:

Child… People come to see me. I cannot treat myself – but people come to me for help. And God helps them- he does it – not me. It is not my miracle; it is His. It is my job to try and see what I can do. There are some people who try so hard that they become closer to God because of their effort. You know that – that whatever effort you make in this regard you will become closer [15]

He suggests that when people come to him for help it is God, *‘not me’,* who helps them, privileging the role of God in determining health outcomes, rather than the skills of any doctor, healer or medicine. Also note the mention of ‘try(ing) and ‘effort’ (twice). This reference to trying also appears in the following quote from this 70-year-old retired factory worker as he expresses gratitude for his illness as well as his faith in God’s divine plan:

I just thank God. That he gives illness because even illnesses come from him. You just keep praying and he keeps listening. Just think – if you have something of your own – would you ever ruin it? No. We are all his. We need to try and he will make it better for us [11]

Personal efforts and faith are both viewed as complementary in the Muslim tradition, where there is a belief that “*there is no disease that Allah has created, except that He also has created its treatment” (*Sahih Al-Bukhari, book 71, Hadith 582, narrated by Abu Huraira). Muslims widely believe that God has created a world where illnesses have ‘treatments’, and that an effort to seek treatment whilst maintaining patience and gratitude is an appropriate response to illness.

Finally, despite the frequent talk of faith and the deterministic view of Allah’s intervention, no participant questioned the technical efficacy of medicines, nor the expertise of the doctors who prescribed them, who were positioned as mediators of God’s will. The ‘active ingredient’ was—in this sense – perceived to be external to the medicines themselves and not directly related to their chemical properties. Rather ‘efficacy’ was constructed as arising from a complex and mysterious relationship between God, patients, medical professionals, and medicines with the patient’s role in taking medicines an expression of ‘trying’ to uphold this constellation. This relationship was succinctly summarised by one participant as follows: ***‘****I ask God for my medicines and my doctors deliver them to me’.* The drugs are available, and the doctors can act as intercessors to guide, advise, and prescribe – but only with the will of God.

### Medicines as a ‘force for good’

Given the faith that Muslim patients express for God’s divine plan, it is unsurprising that many of our participants spoke positively about using medicines, positioning them as important to living their lives and maintaining health. Medicines were identified as a ‘force for good’, powerful intercessory objects enabling them to *‘stay fit’* and be a *‘healthy person’*:

Shall I tell you one thing, it could be that maybe because I take my pills properly and I eat well and take my injections. For that reason, I am fit otherwise I would not be able to stay fit [4]

Interviewer: How did you feel at that time when suddenly you had to take many medicines?

3: I didn’t mind, wanted to have good health. I always wanted to be a healthy person

Note how this 72-year-old female participant describes medicines as the answer to her ‘*pain or problems’*:

14: For my problems, I would need to take medicines. I have just told you my skin is hard, and it is itching. I put some cream, but it didn’t work, when I go to the doctor and tell them my problem, they will give me something. If I want my pain or problems to go then I will need to take another medicine…

Interviewer: You also mentioned that you never miss a medicine.

14: No, I never miss my medicines…As far as I know, if I do not take my medicines, my problems will increase. So why would I leave it?

This participant explains how medicines are the solution to her *‘problems’* on three separate occasions within this short quote. The need to take additional medicines for non-resolving issues, on top of her polypharmacy, is also normalised (‘*If I want my pain…to go… I will need to take another medicine’*). This also implies the belief that there will eventually be a successful medication that works for that *‘problem’*; reflecting the Islamic viewpoint that there is a treatment for all ailments. Finally, she suggests that if she does not take her medicines, her ‘*problems will increase’*- implying her medicines have a role in keeping additional problems at bay.

Our interviews furthermore suggest that people have a belief in medicines as objects. This participant explains how in Pakistan, patients frequently bypass doctors and buy medications directly from the chemist:

(In Pakistan) The pharmacists – well with them you don’t even need to go to the doctor – they have their own store and everything… If you go to the doctor you have to pay two or three hundred rupees, here you can be done in fifty, sixty rupees. It’s a big difference in cost and it seems like they are doing the same thing! The pharmacist has their own medicines, tablets, injections, everything, and they know how much to give of everything. They have all sorts of medicine [11]

The nature of the healthcare system in Pakistan means that for many, buying medicines from chemists directly is financially more viable than seeing a doctor. The role of the doctor in the prescription is thus potentially context-dependent, reliant on balancing cost, availability, and potential trust in the medical system itself (‘*In Pakistan, you know what (doctors) are like…They just want your money’* [11]). All the participants in this study were Pakistani migrants and as such, had some experience with healthcare in Pakistan where access to and affordability of medication is a significant constraint.

Participants told stories which highlighted medicines as a force for good, expressing gratitude that they could access them relatively easily in the UK. Take this quote from one female participant who was the victim of domestic violence, who fled Pakistan and did not return:

Some people just want their doctors to write them medicines and then bin them. I don’t know why they would do such harm. It is harm isn’t it… They should think about how much it costs… it makes me angry. Obviously it makes me angry. The situations I have been in myself (in Pakistan). My kids were so sick and I didn’t have any money. I didn’t have 3 rupees to get my child the injection they needed. Then you get angry that people waste medicine. There are people in Pakistan who do not have one rupee to spare who are desperate for medicines …Here, thank God, medicine is free, doctors are free, hospitals are free [2]

She spoke emotively about people who waste medicines, reflecting on her previous perilous living situation in Pakistan (‘*I didn’t have 3 rupees to get my child the injection they needed’*)—a rhetorical move which highlights the ‘*’harm’* that is done by people who ‘*bin’* their medicines. Medicines are identified by this participant as a force for good that she is now privileged to have *‘free’* access to. We conclude our presentation of findings with this extract from the first author’s reflexive journal.

## Discussion

Polypharmacy is a pressing concern for healthcare systems and disproportionately affects those who are older and from minority backgrounds. To our knowledge, this is the first study exploring polypharmacy in the older British Pakistani community from a generalist perspective. Whyte et al. reflect on the importance of looking at “the lives medicines have *with people* and *between people*” [22]. Our findings suggest interpersonal relationships between patients, doctors, and families in tandem with socio-material relationships between people and medicines are key to understanding how polypharmacy is created and sustained in this population. Moreover, these relationships must be understood as situated and playing out within a shared context of faith. It is only when one contextualises our participants’ narratives within the Islamic and Pakistani cultural paradigms that some of the apparent ambiguities and contradictions that they express can be more fully appreciated. Research which offers patients the opportunity to articulate their experience with medicines in *their own language (*from both a linguistic and cultural perspective) is essential to inform culturally competent and inclusive clinical practices of medicines optimisation.

Existing evidence suggests that people see prescribing as an important role of a doctor in the context of a wider culture of medicalisation where there exists a ‘pill for every ill’ [40]. Research has shown that patients who expect to receive a prescription are more likely to receive one and that Asian patients are more likely to expect prescriptions for certain drugs from their GP than White patients [41, 42]. In the context of a Muslim community, doctors are viewed as moderators through whom Allah might enable effective treatment (or not). The role of the ‘good’ patient is thus to actively ‘try’ and have faith in this possibility. The expectation for a prescription as an important part of clinic encounters was evident throughout our findings—with participants identifying the prescription as the ‘point’ of visiting a doctor and the marker of a ‘good’ doctor. The Islamic belief that there exists a treatment for all diseases is also important. It is not difficult to see that this orientation can support and uphold significant prescribing activity, in that the doctor is invited into a kind of social contract that is both an expression of faith and an occasion of medicine provision. Scholars have described prescribing as “a symbolic act; an effective style of communication… through the prescription, the doctor can tell the patient he understands his problem… the prescriptions is (thus) a proof of the doctor’s concern and competance” [22]. Within time-limited primary care consultations with potential socio-cultural differences, the importance of the prescription as a tool for communication must not be underestimated.

It is estimated that up to 50% of prescribed medications for long-term conditions are never taken [43]. This is thought to be more marked in patients from ethnic minority backgrounds and in the context of socio-economic disadvantage [44–46]. May et al. note that chronic patients are managing not only symptom burden, but also experiencing the “burden of treatment itself, as they engage with services and therapeutic modalities aimed at conditions that cannot be cured but must instead be *managed*” [36]. Research investigating medication management in older people indicates that this daily burden is often hidden from practitioners [47]. Despite all the participants in this study being prescribed more than ten regular medications, participants rarely discussed ‘non-adherence’ or described difficulties taking medicines during our interviews, even when asked directly. In Britten’s study on London General Practice, it was noted that patients rarely discussed their rejection of their medicines with their doctors openly, and even critical patients would be ‘passive’ in medical consultation whilst being more candid in the sphere of everyday life [48]. Whilst we conducted our research in participants’ homes, participants were recruited and contacted initially by their GP. Despite assurances of confidentiality, we cannot rule out the possibility that this approach by their GP may have contributed to participants being unwilling to discuss difficulties with their medications or ‘non-adherence’, a potential weakness of this study.

The formation of narratives has been described as a ‘moral performance’ [49]. Constructions of responsible, compliant, grateful and fundamentally good Muslim patients were evident throughout our interviews. Hawking et al. have reflected on the construction of adherent patient narratives “through dialogue at the intersection of discourses including the authority of doctors, personal responsibility for health, scarcity of resources, and deservingness”… (noting that) the notion of medication adherence places a hidden moral and discursive burden of treatment on patients which they must negotiate when invited into conversations about their medicines” [50]. The interviewer in this study [NS] was of Pakistani background and wore a headscarf, thus was visibly Muslim. Arguably the ‘insider’ status of the interviewer may have shaped our participants’ narratives, giving significance to some storylines over others. It is a particular strength of this study that the shared context of the interviewer invited participants to discuss faith and Pakistani culture openly, culminating in narratives that are rich in spiritual and cultural meaning. The presence of a Muslim interviewer conversely may have heightened the ‘discursive burden’ on participants, to prioritise accounts of themselves as faithful, responsible, and moral members of the Muslim Ummah (Arabic: Community), a shared group to which both interviewer and interviewee belonged. Further limitations of this study include an absence of discussion about medically unexplained symptoms and somatic presentations, which the literature suggests are particularly prevalent in this community and can be associated with inappropriate prescribing [51]. Attitudes to deprescribing were also not discussed explicitly in our interviews and would be important for further work in this population.

Decision making in medical care is understood to be distributed across time, people and situations [52]. Negotiating pharmaceutical decision-making in polypharmacy has been noted as requiring a navigation of tensions across and between system networks (health-care professionals) and life-world networks (family and friends) [53]. It was clear that for the older Pakistani patients in our study, much of the decision-making and work around obtaining and administering medicines is done in close collaboration with family networks. This had implications for how medications were prescribed and obtained, whether they were taken or not, and how they were supplemented with other health-seeking behaviours. Farhat Moazum, an American physician who returned to work in Pakistan following training in the US reflects that “when illness strikes a member of the family in Pakistan it is the family rather that the patient who takes centre stage in this process… the “doctor sahib,” (sahib has an Arabic root meaning “lord”) remains the authority in matters relating to disease … is often symbolically inducted into the family and is expected to direct rather than just facilitate medical management. In the final analysis, however, God, not man, controls life and death”. She goes on to explain that “in (Pakistan’s) deeply religious society, morality is rooted primarily in what is perceived as the religious obligations of the family and the physician toward the patient rather than stemming from a secular, reason-based philosophy that emphasizes the legal rights of individuals” [54].

Finally, our research showed that participants had an emotional attachment to their medications as a ‘force for good’ in their lives. In our interviews, access to medicine, and their ‘good’-ness were seen as an extension of God’s divine will. In Moen et al.’s Swedish focus group study of medication challenges in older Swedish adults, participants “expressed gratitude that medicines exist and felt very fortunate, living rich lives despite diseases and medicine use… The participants (furthermore) had great faith in and respected ‘good’ doctors’ opinions. ‘Good’ doctors were characterized as those taking initiative, listening and giving “the right treatment” … (finally the participants) acknowledged the importance of *having faith* for the treatment to work”. It is interesting to note that whilst this study was done in the Western population, our findings resonate closely with those in this study. Whilst *‘having faith’,* may be important to many populations, the *‘absolute faith’* that our Muslim participants expressed (almost) universally in our interviews was an important finding. For our participants, faith encompassed all aspects of health and illness and steadfastly defined how relationships with doctors and families were enacted. The overarching role of Islam in how our older Pakistani patients made sense of and managed polypharmacy was powerfully expressed throughout our dataset, a faith which functioned as an “anchor” through health and ill-health throughout life.

### Implications for practice and further research

Our findings have relevance to clinicians who care for the British Pakistani community and potentially British Muslims more widely. The themes discussed have potential applicability beyond the issue of polypharmacy, to how care is undertaken more broadly in the older adults in this community. These findings also hold value to academics and policy makers who seek to develop culturally competent prescribing and deprescribing policies in this community. This research finally highlights the need for ongoing scholarship into how patients from minority communities are making sense of and managing their health and care from a generalist perspective.

## Conclusions

Medicines and the bonds that support medication use are materially, socially, spiritually and emotionally powerful. Our research highlights the potential importance of interpersonal and socio-material relationships in how some older Pakistanis understand and manage polypharmacy. Clinicians must better understand the way medication functions as a tool for communication and a symbol of these wider commitments to be able to have meaningful conversations with patients about their medicine. Clinicians also need ways of addressing the moral tensions around medicines in consultation, where the expectation to prescribe can be difficult to navigate in the face of time constraints or in the context of socio-cultural barriers, expectations, and norms. Engaging with the cultural and moral values that patients ascribe to their medicines is likely to be crucial to the success of programmes aimed at medicines optimisation in this community.

## Data Availability

The raw datasets are not available due to the identifiable nature of the research.
